# Seed and Germination Characteristics of 20 Amazonian Liana Species

**DOI:** 10.3390/plants2010001

**Published:** 2013-01-07

**Authors:** Mareike Roeder, Isolde D. K. Ferraz, Dirk Hölscher

**Affiliations:** 1 Xishuangbanna Tropical Botanical Garden, Chinese Academy of Sciences, Plant Geography Lab, Menglun, Mengla, Yunnan 666303, China; 2 Instituto Nacional de Pesquisas da Amazônia (INPA), Coordenação de Pesquisa em Silvicultura Tropical, Av. André Araújo, 2936, Aleixo, Manaus, AM, Brazil; E-Mail: iferraz@inpa.gov.br; 3 Tropical Silviculture and Forest Ecology, Burckhardt Institute, Georg-August-Universität Göttingen, Büsgenweg 1, 37077 Göttingen, Germany; E-Mail: dhoelsc@gwdg.de

**Keywords:** alternating temperature, desiccation, moisture content, photoblastism, seed coat ratio

## Abstract

Lianas are an important component of tropical forests, and may reach their highest densities in disturbed areas. However, information on seed and germination characteristics is scarce. Twenty Amazon liana species were screened for their germination characteristics, including light dependence, tolerance of desiccation and of alternating temperatures; these characteristics are considered important for the germination success in areas with relatively open canopies. Between 31–1,420 seeds per species were available, as 15 species seeds came from one mother plant. We studied seed biometry and conducted germination trials with fresh seeds (12 h light daily, or dark) and desiccated seeds at 25 °C. Germination at alternating temperatures (20/30 °C, 15/35 °C) was analyzed for nine species. Of the 20 species, eight species with the largest seeds had desiccation sensitive seeds; this is the first record for species of four genera and one family, where only desiccation tolerant seeds are otherwise recorded. Light-dependent germination was found in three species (0.01–0.015 g) and is the first record for two; however, results were based on seeds from one plant per species. Alternating temperatures of 15/35 °C decreased final germination of four out of nine species, and response to 20/30 °C cycles varied compared to constant 25 °C. Seed and germination characteristics of the species ranged from pioneer to climax traits indicating that establishment of lianas from seeds may be confined to species specific niches.

## 1. Introduction

Lianas are defined as woody climbers, which germinate on the ground, stay rooted throughout their lives and use other plants as structural support [1]. Due to this growth form, they invest few resources into structure and more in leaf and reproductive biomass [1]. Lianas influence the dynamics of forests in various ways: they contribute to forest species diversity and provide habitat and food for canopy insects and primates [2,3]. By connecting crowns, lianas create pathways for arboreal animals, but also tear down neighboring trees in cases of host-tree fall, and are therefore a concern for forest management. They compete with trees for nutrients, water and light: they can decrease tree growth and fecundity [4,5,6], and may even increase the mortality of trees [7]. Beside edible parts, fibers or ornamental usage, secondary compounds in many liana species are of interest for human use as medicine or poison [8].

Most liana studies over the last decades focused on stem surveys, and information about regeneration and seed ecology remains scarce [9,10,11,12]. Like other woody plants, lianas are able to regenerate by seeds or by sprouting from roots. Additionally, most lianas can survive host tree fall and branch detachment [4], and can subsequently produce a lot of ramets rapidly by sprouting from the stem on the ground and growing laterally into gaps. Reproduction by seeds is still important, as it secures genetic diversity, increases the dispersal area of a species, and in the case of dormant seeds, could overcome unfavorable environmental conditions with time. High densities of lianas occur mainly in naturally or anthropogenically altered areas, such as tree fall gaps or secondary forests, and lianas probably benefit from increasing areas of altered forests [7,13,14,15]. With the increase of secondary regrowth the importance of regeneration by seeds will change: following slash and burn disturbance, succession by newly dispersed seeds will be more important than in areas where vegetation has been slashed without burning and where lianas can resprout from fallen stems [16]. Compared to the more stable environmental conditions in the understory of primary forests, the sparse canopy cover of disturbed forest areas will favor the regeneration of species with seeds that can cope with higher fluctuations of temperature, high irradiation [17] and desiccation. Seed traits are linked to other life history traits of plants, e.g., larger seed size is related to higher seedling establishment under adverse conditions like shading or competition (summarized by [18]). The successional stage of lianas is linked to seed size: early successional communities consist of mainly of small seeded species [19]. Knowing the seed traits and germination characteristics of species helps to estimate the performance of species in different habitats.

Biometric and germination data have been intensively collected for non-cultivated seeds in temperate regions and also for many tree species in the tropics (e.g., Seed Information Database of Kew Garden, [20]). Yet, for other growth forms in tropical forests little information exists. Useful information on seeds can be obtained from very few seeds. For example, Pritchard *et al*. [21] suggest a protocol of only 100 seeds for testing desiccation tolerance of rare species, where exploitation of seeds could be critical for the survival of the population. Daws *et al*. [22] developed a model to predict desiccation tolerance or sensitivity of seeds based only on biometric data. In our case, it was not species rareness but seed availability that restricted the number of studied seeds and species. The aim of this study on liana species of the Central Amazon, Brazil, is to provide trait data related to seeds and seed germination including desiccation tolerance, seed size, seed shape, light dependence of germination (photoblastism), and the influence of alternating temperatures on germination. For several species, we firstly present data on seed traits based on small sample sizes, which could be validated in the future by other studies. This information on traits could be used directly for seed storage of the species, but it mainly provides a base for any ecological study involving seed data, e.g., predicting the success of species establishment in changed landscapes, including clearings or secondary regrowth.

## 2. Results and Discussion

### 2.1. Species with Sufficient Replicates from Various Mother Plants

Sufficient seed numbers were obtained from three to five mother plants (Table 1) of five very common species (*Anemopaegma oligoneuron*, *Arrabidaea trialii*, *Mimosa guilandinae*, *Passiflora nitida*, *Senna* sp. 2) for an analysis with statistically meaningful replicates. These species are mainly found in disturbed areas or secondary regrowth [23]. For all other species seeds were only obtained from one mother plant and were therefore pseudoreplicates or had a very small number of replicates (Table 1). The five common species all had desiccation tolerant seeds; as was revealed in the germination test and the probability calculation for desiccation sensitivity P(D-S) following Daws *et al*. [22], which is based on the seed coat ratio and seed mass; all P(D-S) values were <0.5. Light was not necessary for germination: the relative light germination (RLG, [24]), which is the ratio of germination in light and the sum of germination in light and darkness, was around 0.5 (Table 2). Alternating temperatures inhibited germination in *P. nitida*, but did not affect the other four species (Table 1). Except *P. nitida*, all species had germinated seeds within two weeks. The range of seed mass varied only five-fold, and seed shape was mainly flat or oval-flat, three species had winged dispersal units or seeds (Table 2). The results of these five species will be discussed in context with the other studied species.

### 2.2. Seed Biometrics and Germination at Constant Temperature (25°C)—All Species

A wide variety of morphological traits and physiological responses to environmental factors were found among the 20 study species. The range in dry seed mass covered three orders of magnitude from 0.0085 g (*Matelea badilloi*) to 11 g (*Mucuna* sp.). The length of the longest seed axes (wingless species) differed eight-fold in the extremes, from 5.1 mm (*Cissus sicyoides*) to 43.2 mm (*Gnetum nodiflorum*, Table 2). Seed moisture content at dispersal for all species ranged between 13–68% and was not significantly correlated to dry seed mass (Pearson’s *R* = 0.22 *P* = 0.45, data of seed mass log transformed).

**Table 1:** List of 20 Central Amazonian liana species, the register number at the herbarium at Instituto Nacional de Pesquisas da Amazônia-INPA (Manaus, BR), their botanic family, final germination under various treatments and replicate numbers.

Species with replicates	INPA herbarium number	family- subfamily	Treatment						Replicates x seed number per treatment	mother plants used for seed sampling
			fresh seed				dried seed			
			25 °C L	20/30 °C L	15/35 °C L	25 °C D		25 °C L		
***Anemopaegma oligoneuron* (Sprague & Sandw.) Gentry **	231927	Bignoniaceae	96 ± 8a	98 ± 5a	92 ± 10a	71 ± 24		66 ± 18	25 × 10	5
***Arrbidaea trailii* Sprague **	231926	Bignoniaceae	96 ± 6a	100 ± 0a	94 ± 9a	92 ± 9		86 ± 8	4 × 16	3
***Mimosa guilandinae var. spruceana* (Benth.) Barneby **	231932	Fabaceae-Mimosoidae	32 ± 19a	33 ± 21a	35 ± 22a	23 ± 12		36 ± 23	10 × 20	5
***Passiflora nitida* HBK **	231937	Passifloraceae	20 ± 22a ***	**0 ± 0b**	**0 ± 0b**	14 ± 8		6 ± 7	8 × 25	4
***Senna* sp.2**	231922	Fabaceae-Caesalpinioideae	100 ± 0a	100 ± 0a	98 ± 4a	94 ± 8		89 ± 6	9 × 12	3
Species with pseudoreplicates or small replicates										
*Acacia multipinnata* Ducke		Fabaceae-Mimosoidae	80	80	90	70		40	1 × 10	1
*Anemopaegma floridum* Mart. ex DC	231931	Bignoniaceae	94 ± 9	94 ± 9	100 ± 0	38 ± 0		0 ± 0	2 × 10	1
*Anomospermum solimoesanum* (Moldenke) Krukoff& Barneby	231935	Menispermaceae	90 ± 14	40 ± 30	95 ± 11	44 ± 11		0 ± 0	5 × 4	3
*Aristolochia silvatica* Barb. Rodr.	231928	Aristolochiaceae	**90 ± 10a**	**88 ± 16a**	0 ± 0b **	0 ± 0		72 ± 8	3 × 20	1
*Cissus sicyoides* L.	231923	Vitaceae	93 ± 2a	100 ± 0a	95 ± 6a	25 ± 8		67 ± 30	4 × 25	1
*Coccoloba* sp.	231936	Polygonaceae	**46 ± 13a**	**44 ± 20a**	5 ± 4b *	41 ± 8.5		0 ± 0	4 × 20	1
*Gnetum c.f. nodiflorum* Brongn.	231933	Gnetaceae	7 ± 12	13 ± 11	0 ± 0	0 ± 0		0 ± 0	3 × 5	1
*Matelea badilloi* Morillo	231924	Apocynaceae-Asclepiadoideae	**61 ± 20a**	**98 ± 3b**	0 ± 0c ***	0.6 ± 1.6		63 ± 34	6 × 25	1
*Mucuna* sp.	231940	Fabaceae- Fabaoidae	67 ± 0	na	na	na		0 ± 0	2 × 3	1
*Passiflora c.f. acuminata* DC.		Passifloraceae	43 ± 61	na	na	43 ± 33		20 ± 19	2 × 15	1
*Paullinia c.f. capreolata* (Aubl.) Radk	231939	Sapindaceae	100 ± 0	100 ± 0	100 ± 0	95 ± 1		0 ± 0	2 × 20	1
*Paullinia rugosa* Benth. ex Radk	231938	Sapindaceae	100 ± 0	na	na	95 ± 7		0 ± 0	2 × 10	1
*Smilax* sp.	231930	Smilacaceae	60	na	na	60		0	1 × 10	1
*Strychnos c.f. amazonica* Krukoff	231925	Loganiaceae	50	na	na	70		0	1 × 10	1
*Strychnos glabra* Sagot ex Progl.	231929	Loganiaceae	65 ± 7	75 ± 7	25 ± 0	65 ± 7		0 ± 0	2 × 10	1

Germination trials were conducted at 25 °C constant temperature or 12 h/12 h alternating temperatures of 20/30 °C and 15/35 °C with daily 12 h photoperiod (L), in complete darkness (D) at 25°C and after desiccation (25 °C). Final germination percentages (mean ± SD) are given. For species with ≥3 replicates and ≥60 seeds per treatment, statistical differences of the treatments 25 °C, 20/30 °C, 15/35 °C are indicated in lowercase letters (Kruskal-Wallis-Rank Sum-Test with subsequent pair-wise Wilcoxon-Test). Significance levels * *p* < 0.05, ** *p* < 0.01, *** *p* < 0.001. *A. silvatica*: significance of ANOVA, as replicate number was only three (*F* = 16.1, df = 2, *p* > 0.004). Species names are according to TROPICOS [25].

**Table 2:** Seed and germination characteristics of 20 liana species ordered by increasing dry seed mass.

Species with replicates	fresh seed mass (g)	n	seed moisture (%)	n	longest axis (mm)	n	seed shape	P (D-S)	n	G (DT-DS)	RLG	germination time (d)			
												1st	50%		
***M. guilandinae***	0.016 ± 0.005	30	13.2 ± 5.2	30	7.0 ± 0.9	60	flat	0.055	20	DT	0.58	5	9		
***Senna* sp. 2**	0.031 ± 0.008	50	34.6 ± 3.3	50	6.4 ± 1.0	50	oval	0.002	20	DT	0.52	2	2.5		
***P. nitida***	0.033 ± 0.006	50	32.0 ± 3.4	50	7.0 ± 0.7	50	flat	0.001	20	DT	0.58	108	156		
***A. trailii***	0.035 ± 0.008	41	29.9 ± 8.8	41	29.2 ± 4.4	41	winged & flat	0.037	20	DT	0.51	11	23		
***A. oligoneuron***	0.191 ± 0.042	60	65.2 ± 3.6	60	38.7 ± 4.3	60	winged & flat	0.026	23	DT	0.57	14	16		
Species with pseudoreplicates or small replicates															
*M. badilloi*	0.016 ± 0.002	50	48.4 ± 2.9	50	8.9 ± 0.4	50	flat	0.001	20	DT	0.99	5	15		
*P. acuminata*	0.021 ± 0.005	15	47.6 ± 11.6	14	7.3 ± 0.3	30	flat	0.017	18	DT	0.68	92	105		
*C. sicyoides*	0.031 ± 0.002	30	31.1 ± 3.2	30	5.1 ± 0.3	30	drop like	0.010	20	DT	0.79	11	24		
*A. multipinnata*	0.037 ± 0.008	30	24.2 ± 3.5	14	6.4 ± 0.7	30	flat	0.068	21	DT	0.53	3	3		
*A. silvatica*	0.035 ± 0.004	30	30.4 ± 3.2	30	18.6 ± 1.9	30	winged & flat	0.022	21	DT	1.00	10	84		
*A .floridum*	0.396 ± 0.037	30	65.0 ± 3.7	10	52.9 ± 4.8	30	winged & flat	0.060	15	DS	0.71	21	31		
*Coccoloba* sp.	0.238 ± 0.051	51	40.5 ± 3.8	51	7.7 ± 0.6	51	round	0.222	20	DS	0.53	31	70		
*P. rugosa*	0.464 ± 0.097	30	42.3 ± 3.9	10	10.0 ± 0.8	30	round	0.780	7	DS	0.51	9	12		
*S. amazonica*	0.691 ± 0.221	30	52.1 ± 3.1	11	14.9 ± 1.8	30	triangular	0.523	9	DS	0.51	20	44		
*S. glabra*	0.776 ± 0.101	30	56.2 ± 3.1	10	12.4 ± 0.7	30	triangular	0.814	9	DS	0.42	20	27		
*Smilax* sp.	0.584 ± 0.123	30	41.6 ± 3.1	10	11.3 ± 1.2	30	triangular	0.770	7	DS	0.50	20	29		
*P. capreolata*	0.477 ± 0.051	10	28.9 ± 3.1	10	13.3 ± 0.5	30	oval	0.814	10	DS	0.50	4	6		
*G. nodiflorum*	12.19 ± 2.346	46	50.2 ± 3.4	19	43.2 ± 3.1	46	oval	0.977	12	DS	1.00 n = 1	155 n = 1	155 n = 1		
*A. solimoesanum*	13.97 ± 2.603	84	32.0 ± 3.2	22	36.1 ± 2.6	84	oval	0.543	20	DS	0.67	91	157		
*Mucuna* sp.	34.16 ± 12.002	25	67.8 ± 3.2	9	38.8 ± 5.7	25	round	0.984	10	DS	na	53 n = 4	60 n = 4		

Given are mean ± standard deviation and sample size (n), dessication tolerance (DT) and dessication sensitivity (DS) based on germination trials (G) and probability of desiccation sensitivity (P) calculated after Daws *et al.* [22], relative light germination (RLG) determined after Milberg [24], time for germination of 1st seed and of 50% of germinable seeds.

Amongst all species, the time until the first seed showed radical protrusion at the constant temperature of 25 °C was between two and 155 days; in a similar time range 50% of all germinated seeds had developed a radical (2–157 days, Table 2). No meaningful pattern in germination velocity related to biometric data could be found. The type of dormancy that delayed germination was not determined in this study. For *P. nitida*, which was one of the slowest germinating species in this study, a dormancy period of several months and low final germination are known [26].

For eight species final germination of the seeds at constant 25 °C with light was ≥90%. The lowest final germination was found in *P. nitida* (20%) and *G. nodiflorum* (7%) (Table 1). Seed viability of all seeds at the end of the germination trials (excluding the desiccation treatment) was >91% for six species, 78–88% for four species (*Coccoloba* sp., *M. badilloi*, *M. guilandinae*, *Strychnos glabra*), and two species could not be tested (*P. nitida*, *G. nodiflorum*).

### 2.3. Desiccation Tolerance—All Species

**Figure 1:**
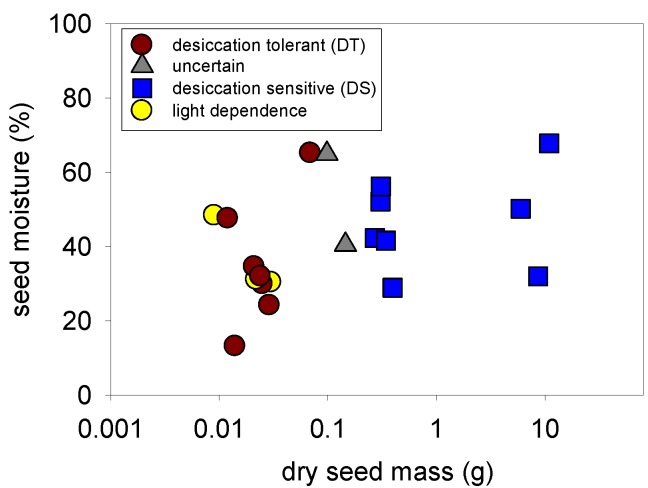
Seed moisture at dispersal of 20 liana species of the Central Amazon in relation to seed size (dry mass) with indication of desiccation tolerance or desiccation sensitivity of the seeds and necessity of light for germination (photoblastic seeds). Note log-scale at x-axis.

We assessed desiccation tolerance using two techniques (germination trials and probability equation based on biometric data [22]), which should add reliability to our results. In fact, results of both techniques were consistent for 18 out of 20 species (Table 2, Figure 1). The ten species that have smaller seeds (up to 0.07 g dry seed mass) germinated after desiccation to a moisture content <9% (3.7–8.8%) and had a calculated probability of desiccation sensitivity P(D-S) < 0.5 (Table 2). These species all belong to families or genera with seeds that are known to be tolerant to desiccation, and species records exist for *Cissus sicyoides* [20]. An intermediate desiccation tolerance (seeds tolerate desiccation not below 5% moisture content, neither temperatures below 0 °C, [27]), as found in some *Passiflora* species, e.g., *P. edulis* [28], could not be excluded for *Passiflora acuminata or P. nitida* in our experimental design. 

Eight species with larger seeds (dry seed mass > 0.27 g) and P(D-S) > 0.5 (*Anomospermum solimoesanum*, *G. nodiflorum*, *Mucuna* sp., *Paullinia rugosa*, *Paullinia capreolata*, *Smilax* sp., *Strychnos amazonica*, *S. glabra*) did not germinate after desiccation and seeds were dead. This corresponds with the finding of Hong and Ellis [29] that the percentage of species with desiccation sensitive seeds increases with increasing seed size.

Desiccation sensitive seeds have been recorded for *Paullinia cupana* (Sapindaceae) [30], a well studied species of economic importance in the Amazon region, which supports our finding in the same genus for *P. capreolata*. There are no records of desiccation sensitive seeds for the genera *Smilax*, *Strychnos*, *Gnetum* and *Mucuna*, but there are records of some desiccation tolerant congeners [20,31]. No seed information was found for the genus *Anomospermum*, the few species investigated from the same family (Menispermaceae) all had desiccation tolerant seeds [20]. The mentioned records of desiccation tolerant congeners often included smaller seeded species and some are from temperate regions. Desiccation sensitive seeds are most common in the tropics, and, therefore, the probability of finding a species with desiccation sensitive seeds of a certain genus or family is higher in tropical than in temperate regions [32].

Seed shape was correlated to the probability of desiccation sensitivity: flat seeds all had low P(D-S) values, rounder seeds had a wider range of probability values; but overall the correlation was positive (Spearman’s rho = 0.62, *p* = 0.003). Hong and Ellis [33] found seed shape is associated with desiccation tolerance within the Meliaceae family: desiccation sensitive species being round, desiccation tolerant species being generally flat.

Another trait related to desiccation sensitivity is seed moisture content at dispersal [33]. However, no relationship between moisture content and desiccation sensitivity was found in the tested species. As seeds were weighed immediately after collection or extraction from the fruits, fresh weight and moisture content of the winged or hair tuft (coma) bearing species (*A. oligoneuron*, *Anemopaegma floridum*, *A. trailii*, *Aristolochia silvatica*, *M. badilloi*) might be slightly overestimated, as these seeds would probably dry some hours or days later in the opened fruit before wind dispersal. In the case of *Senna* sp. 2 (35% moisture content), it can be assumed that seeds were still water permeable during collection and might have dried more after dispersal. Hong and Ellis [29] also mentioned that predicting desiccation tolerance is not possible for seed moisture content in the range of 25% to 55% at dispersal time. Desiccation sensitive seeds are expected to shed during the rainy season to avoid any risk of severe water loss. In our study, seeds of five desiccation sensitive species were shed at the end or beginning of the rainy season (June, July and November) and three shed during the rainy season (April, May). Desiccation tolerant seeds were shed during the rainy season (3 species), the dry season (4 species) at the transition from wet to dry season (2 spesies), and also over several months (3 species).

Two species (*Coccoloba* sp. and *A. floridum*) revealed contradicting results in the germination test and calculated probability of desiccation tolerance: they did not germinate after desiccation (0% germination) but should be desiccation tolerant according to the calculated probability P (D-S) = 0.22 and 0.06, respectively. The phylogenetic relationship of both species suggests they could have desiccation tolerant seeds; only species with desiccation tolerant seeds are known from the Bignoniaceae family (*A. floridum*) (80 species records, [20]), and for Polygonaceae (*Coccoloba* sp.) so far only one tree with desiccation sensitive seeds (*Triplaris cumingiana*) has been recorded, but 159 species with desiccation tolerant seeds are known [20,34]. The lack of germination after drying might be the result of using immature seeds in the treatments, which may not yet have acquired the tolerance to desiccation at the end of maturation drying [35]. In doubtful cases, the calculation of P (D-S) after Daws *et al*. [22] seemed to be more reliable for determining desiccation tolerance than germination tests, and can also support uncommon findings, e.g., that the *Mucuna* species is desiccation sensitive, where smaller seeded congeners are usually known for desiccation tolerant seeds and hard seed coats [36]. Biometric data may be more robust considering the difficulty in recognizing the state of seed maturation at harvest of species with unknown phenology. 

### 2.4. Light Germination—All Species

Two species (*M. badilloi* 0.009 g, *A. silvatica* 0.03 g) needed light for germination (photoblastic seeds), RLG was ≥ 0.99, as there was no germination in darkness and final germination in light was 61% and 90% respectively. *C. sicyoides* (0.02 g), with 25% germination in darkness and 93% in light, had a RLG of 0.79. The high RLG suggest that seeds of these three species could be considered photoblastic. Replication size was sufficient (at least 3 × 20 seeds), yet results cannot be considered as totally reliable since seeds were harvested from only one liana plant per species. To our knowledge this is the first record indicating photoblastism of *A. silvatica* and *M. badilloi*. Photoblastism of *C. sicyoides* had already been documented in an earlier field study [10]. All three species had seeds with 0.009–0.03 g dry mass. The chance that light is required soon after germination to support growth for photosynthetic self sufficiency is higher in small seeded species with restricted resources than in large seeded species. Thus with increasing seed size, the share of species with light depending seed germination is reduced as shown for different growth forms (herbaceous plants, trees) in tropical and temperate zones [17,24,37].

Four more species had RLG ≥ 0.67 (Table 2), but replicate number (*A. floridum*, *A. solimoesanum*, *P. acuminata*Table 1) and/or germination of the control was very low (*G. nodiflorum*, one seed) and do not allow an affirmation of light dependence of germination.

### 2.5. Germination at Alternating Temperatures

**Figure 2:**
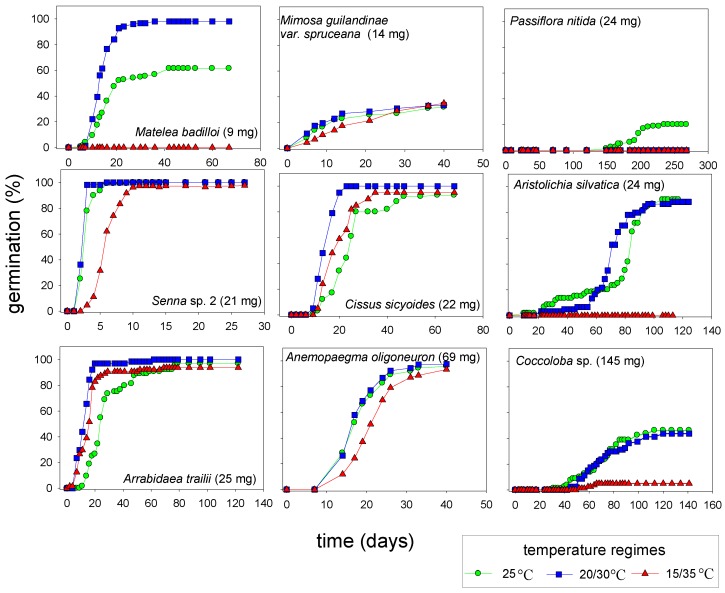
Germination curves of nine liana species of the Central Amazon at constant (25 °C) or 12/12 h alternating temperatures (20/30 °C, 15/35 °C) with 12 h light daily. The species were ordered by increasing seed resources (dry mass of embryo and endosperm), dry mass of whole seed is given in brackets. For significant differences of final germination between temperature conditions see Table 1.

The understory of old growth tropical forests present a habitat with small daily temperature alternations compared to open areas such as gaps [17], borders or secondary forests. In this study, the different environments were represented by a constant temperature and two regimes of alternating temperatures. Germination at alternating temperatures was tested with 14 species. In general, high alternating temperatures (15/35 °C) had no influence or reduced germination; low alternating temperatures (20/30 °C) induced various responses (Table 1). Replicates and seed numbers were sufficient for statistical analysis for only nine species (≥3 replicates, ≥60 seeds per treatment, Table 1, Figure 2). Of these nine species, final germination differed significantly among treatments in four species. *P. nitida* germinated only at constant 25 °C. *A. silvatica* and *Coccoloba* sp. reached the same final germination result under both a constant temperature and small daily temperature cycle (30/20 °C); however, with the larger daily temperature cycles (35/15 °C) germination dropped to 0% and 5%, respectively. Germination increased in only one case (by 1.6-fold in *M. badilloi*, 9 mg dry seed mass) under alternating temperatures of 20/30 °C (Table 1), but no germination occurred under large temperature cycling (35/15 °C). Increased germination at alternating temperatures has also been reported for a related vine, *Matelea maritima* (setting: 25/35 °C, [38]). No related records were found for the other three species. 

In former studies, the influence of alternating temperature on germination was related to seed size: Small seeded species (0.04–0.68 mg) were found to be intolerant to temperature fluctuations whereas for relatively larger seeds (>2 mg) germination rate increased with temperature fluctuations [17]. Ecologically, this intolerance may prevent small seeded species germinating in unfavourable conditions for seedling establishment, e.g., high irradiation and drought in large clearings, though these species still depend on smaller vegetation gaps for seedling establishment [17]. However, no specific pattern of germination response to temperature fluctuations was found with increasing seed resources (embryo and endosperm dry mass) in our study. This was probably because the range of seed sizes in our study did not include species with extremely small seeds (<1 mg).

As summarized by Probert [39] temperature cycles stimulated germination for many species and were often linked to photoblastism. Alternating temperatures may enhance stimulation through light or even enable dark germination of initially photoblastic seeds. However, in the present study one species with photoblastic seeds (*C. sicyoides*) tolerated alternating temperatures and the two others did not (*M. badilloi*, *A. silvatica*); the combination of dark germination and alternating temperatures was not tested here. This indicates that species of the same growth form with photoblastic seeds still differ in their niche of regeneration.

## 3. Experimental Section

### 3.1. Seed Collection and Biometry

Mature fruits and seeds of 20 liana species belonging to 12 families were collected close to their natural time of dispersal in the surrounding area and up to 80 km north of Manaus (3°–2.25° S, 60°–59.5° W). The climate is tropical-humid, with 2,285 mm annual rainfall [40], and a dry season from June to October. Species were identified by comparing specimens at the herbarium of the Instituto Nacional de Pesquisas da Amazônia (INPA), Brazil. Names follow the database TROPICOS [26] (Table 1). Some of the species are subwoody-woody persistent climbers and one is a climbing shrub (*Senna* sp. 2), but they were nevertheless included here, following the liana census of Gerwing [41]. Seed collection was restricted to one plant for most species, but seeds were obtained (used here in a broader sense for diaspore) from three to five individual plants at different time points for six species (*A. oligoneuron*, *A. solimoesanum*, *A. trailii*, *M. guilandinae*, *P. nitida*, *Senna* sp. 2, Table 1). The time between collection and assessment of biometry or implementation of the germination tests did not exceed 10 days, during this time fruits and seeds were maintained at 15 °C. Immediately after extraction from the fruits, where succulent pulp was eliminated if necessary, biometric measurements were made with at least 30 seeds. For two species fewer seeds were available (Table 2). Maximal length, width and height were measured with a digital caliper (0.01 mm). Fresh seeds were weighed individually (0.001g) and subsequently dried until constant dry mass was reached, at 105 °C for 24–62 h (depending on seed size) and weighed again. Seed moisture content was calculated as the percentage of fresh mass. Seed coat ratio (SCR; seed coat mass versus whole seed mass) was determined by dissecting 7–20 dried seeds.

### 3.2. Desiccation Tolerance

Seeds were dried in two sub-samples in an air conditioned room (25 ± 4 °C, 60 ± 10% relative humidity) above a fan between five to eight days until a constant mass was reached. Afterwards seeds were dried for another seven days over the same amount of silica gel as seed mass in three layers of hermetically closed plastic bags. Seed moisture after desiccation was determined for a part of the seeds (n= 4–50), as described above, and the other seeds were re-hydrated slowly for seven days in an atmosphere with saturated relative humidity, avoiding imbibition damage by direct contact to water [18], before the assessment of germination. A species was classified as desiccation sensitive when all dried seeds in the germination test were dead.

### 3.3. Germination Test

Seeds were sown on water saturated vermiculite (fine or medium grain, Eucatex Agro^®^) in transparent plastic containers (11 cm × 11 cm × 3 cm), or in Petri dishes (9 cm diameter) for small seeded species (*Senna* sp. 2, *Acacia multipinnata*) with vermiculite as substrate. The containers were wrapped in loosely closed transparent plastic bags to avoid desiccation. Three temperature regimes were provided, two incubators for each temperature regime were used (FANEM Mod. 347 CDG São Paulo, Brazil and LMS cooled incubator, York, UK): (i) constant temperature at 25 °C with either a 12 h photoperiod (white fluorescent light with approximately 70 µmol m^−2^ s^−1^ PAR) or in complete darkness, (ii) 12/12 h alternating temperatures of 20/30 °C and (iii) 12/12 h alternating temperatures of 15/35 °C, the period of higher temperature coincided with the 12 h light period. The average thermal input for all three temperature conditions was 25 °C. For the dark treatment, the containers were wrapped in two layers of aluminum foil and germination was assessed only after the germination curve under light conditions reached an asymptote, for slow germinating species the substrate was remoistened in dim green light. For one slow germinating species (*P. nitida*), seedlings in the dark treatment died, but were still accurately countable. Dried seeds were only tested at constant 25° C with 12 h light. The number of repetitions and seeds per repetition depended on seed availability and ranged between 2 × 3 seeds to 25 × 10 seeds per treatment (Table 1). Germination in light was assessed after radical protrusion (<1 mm). Observations were made daily, three times per week, or weekly, depending on the germination speed of the species. Seeds that did not germinate were tested for viability at the end of the germination trials via a staining (2,3,5 triphenyl tetrazolium chloride, 1% solution) or cutting test.

### 3.4. Data Analysis

The probability of desiccation sensitivity or tolerance P (D-S) was calculated following Daws *et al*. [22]. This equation (1) includes data of the seed coat ratio (SCR, in formula: a) and the log10 value of the whole seed mass (b). If P (D-S) is > 0.5 seeds are probably desiccation sensitive.


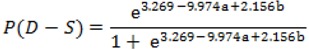
(1)

The requirement for light during germination was calculated by using the relative light germination index (RLG) based on Milberg [30]. The index was calculated as RLG = Gl/(Gd + Gl) where Gl = the germination percentage in light, and Gd = the germination percentage in darkness. RLG = 1 when germination is obligate light depending. The ratio of shortest to longest seed axis was used to create an index for seed shape (1 = round seed, towards 0 = flat seed). 

Differences in germination between control and alternating temperatures were tested via Kruskal Wallis Rank Sum Test and subsequent pair wise Wilcoxon Test. In one case (*A. silvatica*, alternating temperature), ANOVA and subsequent Tukey Post Hoc Test with arcsine transformed values was used instead of Kruskal-Wallis Rank Sum Test, due to small replicate numbers (n = 3). For correlation, Pearson’s correlation was used, or Spearman rank correlation in case of non-normal distribution of data. Statistical analyses were carried out with R-2.13.2 (R Core Development Team 2011).

## 4. Conclusions

The 20 tested species represent perhaps 5% of local liana species. In a reserve (100 km^2^) close to Manaus, around 300 liana species were found [23]. No general conclusion about seeds of lianas can be drawn and the results are often based on seeds of one single plant per species. Nevertheless, given the scarcity of data for some of the species, we provide useful baseline information of germination behavior for any future research on these liana species. The survey of morphological and physiological traits provided redundancy of results, which were especially helpful in cases of low germination success or restricted seed supply. In five species, the results are based on statistically sufficient sample sizes and mother plants.

The growth form of lianas is very diverse; this evidence applies also to seed, seedling and germination characteristics. We identified a range of habits, from typical pioneer species (photoblastic, desiccation tolerant seeds), to climax species (large, desiccation sensitive seeds). The contrasting responses to alternating temperatures in a small range of seed sizes (9–145 mg dry mass), suggests that the establishment of lianas from seeds may be restricted to very species specific niches. First records of desiccation sensitivity of seeds or light depending germination for some species add another stone to the mosaic of knowledge about lianas.

Time restrictions on research projects and the requirement of sound statistics for publications drives seed knowledge towards species with easily accessible, abundant seeds; however, for difficult or rare plant groups (e.g., species with few large seeds, supra-annual fruiting cycles, hard access) several small imperfect data sets may validate each other and should be made accessible.

## Figures and Tables

**Appendix 1:**
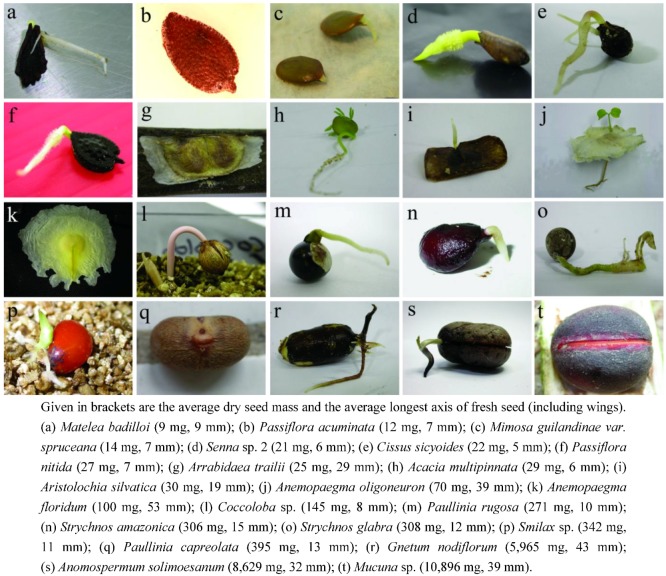
Images of fresh or germinating seeds of all studied liana species.
